# Impact of pathogenic mutations of the GLUT1 glucose transporter on solute carrier dynamics using ComDYN enhanced sampling

**DOI:** 10.12688/f1000research.18553.2

**Published:** 2022-06-13

**Authors:** Halima Mouhib, Akiko Higuchi, Sanne Abeln, Kei Yura, K. Anton Feenstra

**Affiliations:** 1 Laboratoire Modélisation et Simulation Multi Echelle (MSME) - UMR 8208 CNRS, Université Paris-Est, Champs-sur-Marne, France; 2 Institut Universitaire de France (IUF), Paris Cedex, 05 75231, France; 3 Graduate School of Frontier Sciences, The University of Tokyo, Tokyo, Japan; 4 Dept. Computer Science, Integrative Bioinformatics, Vrije Universiteit, Amsterdam, The Netherlands; 5 Graduate School of Humanities and Sciences, Ochanomizu University, Tokyo, Japan; 6 School of Advanced Science and Engineering, Waseda University, Tokyo, Japan

**Keywords:** GLUT1 glucose transporter deficiency syndrome, Human glucose transporters, SLC transporter family, transport mechanism, molecular dynamics simulation, Martini force field, coarse-grained simulations, enhanced sampling method

## Abstract

**Background:** The solute carrier (SLC) family of membrane proteins is a large class of transporters for many small molecules that are vital for cellular function. Several pathogenic mutations are reported in the glucose transporter subfamily SLC2, causing Glut1-deficiency syndrome (GLUT1DS1, GLUT1DS2), epilepsy (EIG2) and cryohydrocytosis with neurological defects (Dystonia-9). Understanding the link between these mutations and transporter dynamics is crucial to elucidate their role in the dysfunction of the underlying transport mechanism, which we investigate using molecular dynamics simulations.

**Methods:** We studied pathogenic and non-pathogenic mutations, using a newly developed coarse-grained simulation approach ‘ComDYN’, which captures the ‘COMmon constraints DYNamics’ between both states of the solute carrier protein. To guarantee the sampling of large conformational changes, we only include common constraints of the elastic network introduced upon coarse-graining, which showed similar reference distances between both conformational states (≤1 Å difference).

**Results:** ComDYN is computationally efficient and sufficiently sensitive to capture effects of different mutations. Our results clearly indicate that the pathogenic mutation in GLUT1, G91D, situated at the highly conserved RXGRR motif between helices 2 and 3, has a strong impact on transporter function, as it blocks the protein from sampling both conformational states. In comparison, predictions from SIFT and PolyPhen only provided an impression of the impact upon mutation in the highly conserved RXGRR motifs, but yielded no clear differentiation between pathogenic and non-pathogenic mutations.

**Conclusions:** Using our approach, we can explain the pathogenicity of the mutation G91D and some of the effects of other known pathogenic mutations, when we observe the configurations of the transmembrane helices, suggesting that their relative position is crucial for the correct functioning of the GLUT1 protein. To fully understand the impact of other mutations in the future, it is necessary to consider the effect of ligands, e.g., glucose, within the transport mechanism.

## Introduction

**Figure 1.**
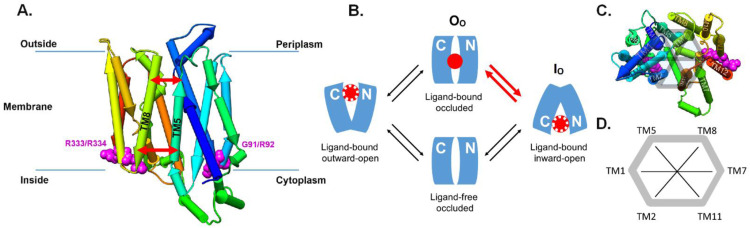
GLUT1 structural overview. ( **A**) Pipe representation of the inward-open (I _O_) conformation (PDB-ID: 5EQI; bound inhibitor removed) of GLUT1 situated in the lipid bilayer. Note that the protein structure has a two-fold rotational symmetry and the two conserved RXGRR-motifs are located at the junctions of the transmembrane (TM) helices 2 and 3 and TM8-TM9, around the R333/R334 and G91/R92 mutation sites shown in magenta. The red arrows symbolize the inside and outside distances. Note that we number the helices starting from TM1 at the N-terminus of the transporter (dark blue in the pipe representation). ( **B**) Schematic cycle of the glucose transport mechanism between the four different open and occluded states as adapted from ( Deng *et al.*, 2015). The bound glucose ligand is indicated as a red sphere. In the open states (inward or outward), glucose may be bound or unbound; this is represented using white dots. This work concerns the first part of the transport mechanism, i.e., the dynamics between the outward-occluded state (O _O_) and inward-open state (I _O_) as highlighted with red arrows. Note however, that we do not consider ligand binding within the scope of this work (see Discussion further below). ( **C**) Pipe representation of the I _O_ conformation (PDB-ID: 5EQI) of GLUT1 viewing on the outward facing part of the transporter inside the periplasm. ( **D**) Definition of the order parameters to follow the motion of the helices over the ComDYN simulations.

The solute carrier (SLC) transporter superfamily is known to play a key role in the transport of small molecules. The superfamily comprises 52 families, and at least 386 different transporter genes have so far been identified in humans ( Hediger *et al*., 2013; Higuchi *et al.*, 2018). This family of membrane proteins is a large class of transporters for many small molecules such as glucose that are vital for the cell, and can be found in all kingdoms of life. Of particular interest are the glucose transporters SLC2A1 from the SLC2 subfamily; GLUT1 mutations are associated with GLUT1 deficiency syndrome (GLUT1DS1 and GLUT1DS2), and some forms of spasticity (Dystonia-9) and epilepsy (EIG2) ( Klepper *et al*., 2016; Mongin *et al*., 2016). Shedding light on the molecular mechanism of the transport function, enables us to understand the difference between pathogenic and benign mutations that have been observed in human subjects. Glucose transporter GLUT1 is built from 12 transmembrane helices (TMs) and exhibits a two-fold symmetry plane joining the two times six TM helices over a bridging helix on the cytoplasmic side of the membrane (see Figure 1A). The transport mechanism of glucose involves cycling through four states ( Deng *et al.*, 2015): outward open (ligand bound or ligand free), outward occluded (O _O_, ligand bound), inward open (I _O_, ligand bound or ligand free), and inward occluded (ligand free), as summarized in Figure 1B. Throughout the SLC transporters, a highly conserved RXGRR-motif is found between TM2 and TM3 and between TM8 and TM9 at the intracellular side of the corresponding loops ( Pao *et al*., 1998; Sato & Mueckler, 1999). Several mutations at these anchor points are known to be disease-related, such as G91D and R92W which are known to cause GLUT1DS1 ( Klepper *et al*., 2001; Klepper & Voit, 2002), whereas R93W is associated with GLUT1DS2 ( Joshi *et al*., 2008). Between TM8 and TM9, R333W is also confirmed to be pathogenic ( Klepper *et al*., 2001; Klepper & Voit, 2002), while the clinical significance of R334Q is unknown, although it is likely to affect the protein function. 

Here, we investigate the effect of different mutations on the dynamic of the human GLUT1 protein. As the dynamic response upon mutation may depend on the conformational state, we aimed to simulate the first part of the transport mechanism between the outward-occluded state (O _O_) and inward-open state (I _O_), as shown in Figure 1B. Hereby, we investigate the intrinsic dynamics of the transporter protein without the ligand. For GLUT1, however, a three-dimensional structure is only available for the I _O_ state; therefore, simulations of GLUT3, which is evolutionarily quite close to GLUT1, in the O _O_ state were analysed. GLUT3 is also suspected to be associated with neurological disorders such as Alzheimer ( An *et al*., 2018; Gu *et al*., 2018; Simpson *et al*., 1994; Szablewski, 2017). We expect that the intrinsic dynamics of the ligand-free states of the transporter provides new insight on the effects of the pathogenicity of certain mutations, without explicit consideration of the ligand-bound states.

## Methods

### Starting structures

The crystal structures of both I _O_ and O _O_ states are available in the PDB; 5EQI for GLUT1 I _O_ state ( Deng *et al*., 2015), and 4ZW9 for GLUT3 O _O_ state ( Kapoor *et al*., 2016); these were used as starting points for the simulations, as we focus on the first part of the transport mechanism. The next step, i.e., to elucidate the complete pathway, would be to extend our work to the outward-open conformation which is also available in the PDB (4ZWC GLUT3 with maltose bound), and to study the effect of ligand binding on the transporter dynamics. However, this is beyond the scope of the present study. Based on the reported pathogenic mutations of GLUT1 in the conserved RXGRR motif region, we searched the corresponding positions of GLUT3 for additional mutations and included them in our study. Due to the high sequence identity (~70%) between the proteins, we intentionally did not build a homology model for one or the other protein. This is justified, as our main aim is to characterize the global opening and closing mechanism rather than to look into atomistic details such as protonation states. Moreover, we now avoid additional uncertainties about details of the structure as would be inevitably introduced during the homology modelling process. Our composite scheme using coarse-grained molecular dynamics with common constraints elastic network ‘ComDYN’, that keeps part of the unchanged position constraints between the two protein structures, is explained in the approach below and further details are supplied in the supporting methods, available in the deposited code ( Feenstra, 2019b).

### Molecular dynamics simulations

In this study, we employed molecular dynamics (MD) simulations using the GROMACS 4.0.5 programme package ( Hess *et al*., 2008). For efficiency reason, we investigated the applicability of the MARTINI coarse-grained (CG) force field ( Arnarez *et al*., 2015; de Jong *et al*., 2013; Hsu *et al*., 2017; Monticelli *et al*., 2008; Periole *et al*., 2009), which is about 500-fold faster than the full-atomistic GROMOS ( May *et al*., 2014). This speed-up is obtained at the expense of explicit description of hydrogen bonds in the protein, which necessitates the addition of an elastic network to maintain secondary structure and other tertiary contacts and thus overall protein stability; the elastic network is thus tailed to a particular conformational state of the protein. Here, we propose to modify the elastic network that is used in MARTINI based on our starting conformations, to include only common elastic network constraints that differed less than 1Å between the I _O_ and O _O_ states. Increasing the distance threshold for the common constraints would result in even fewer constraints in the ComDYN, increasing the risk of instability and possibly unfolding of the protein. Therefore, our chosen constraints allow transition between both states, while maintaining protein structure stability. This composite scheme will henceforward be referred to as ComDYN (COMmon constraints DYNamics). The detailed computational set-up is provided in the Supporting Methods ( Feenstra, 2019b).

### Analysis of transporter dynamics

Essential dynamics analysis was performed on the GLUT1 and GLUT3 simulations using the built-in analysis tools of GROMACS. To allow this comparison between these two homologous proteins, and allow for focusing on overall motions of the transporter region, we selected the structurally conserved helical segments, as summarized in Supporting Table S2, available as extended data ( Feenstra, 2019a). Then, the covariance and eigenvalue calculation were performed on the ensemble of both wild-type systems, using the coordinates of the C-alpha atoms for the full atomistic (AT) simulations, and the backbone particle at the C-alpha position for the coarse-grained (CG) and ComDYN simulations.

To analyse the transporter dynamics from the ComDYN, we defined several order parameters as previously proposed by Nagarathinam *et al.* (2018) by measuring distances between adjacent TM helices, at the intracellular (in)- and extracellular (out)sides of the protein (see Figure 1B, C). For each of the ring of six central helices that make up the solute transporter region, TM2, TM1, TM5, TM8, TM7, TM11 (and back to TM2), we defined an ‘inside’ and ‘outside’ segment of ten residues (See Figure 1 and extended data, Supporting Figure S3 ( Feenstra, 2019a)). Comparing the distances of the mutations to both wild types allows us to capture abnormal behaviour and identify the mutations that have the highest impact on the opening and closing mechanism of the apo-form of the transporter protein (see Results and Discussion).

To compare the distributions of sampled distances during the simulations, two metrics were employed: overlap and shift. ‘Overlap’ is the fraction of overlap between both distributions, as the integral of the minimum of both functions. ‘Shift’ quantifies the direction of change, and is obtained by taking the difference in the position of the peak of the two distributions from the ensemble of the simulations (typically, mutation version wild-type). Negative indicates a ‘closing’ motion, positive is ‘opening’. This analysis was performed using the script ‘calc_overlap.py’, which may be found in the extended data.

## Results and discussion

### Prediction of mutation impact

Table 1 lists the mutants of GLUT1 and GLUT3 that we will consider here, which are situated in the conserved RXGRR-motif distal to the transporter region. The table lists the predicted impact upon mutation obtained from SIFT ( Sim *et al*., 2012) and PolyPhen-2 ( Adzhubei *et al*., 2010). Most mutations are classified as likely pathogenic by both methods, with the exception of GLUT1 R93Q, and GLUT3 R91C and R91H. However, these methods are trained on the dbSNP database which also includes these known mutations, so this should be no surprise. Moreover, these predictions do not allow us to gain any insights into the mechanism by which these mutations may affect transporter function.

**Table 1.** Overview of selected mutations of GLUT1 (PDB-ID: 5EQI) and GLUT3 (PDB-ID: 4ZW9) studied in this work and the impact predictions obtained from SIFT and PolyPhen. The pathogenic mutations in GLUT1 are underlined.

Protein	Mutation	Clinical significance *	SIFT	PolyPhen
GLUT1	G91D	( Klepper *et al.*, 2001; Klepper & Voit, 2002) GLUT1DS1 omim:606777	affect protein function	probably damaging
	R92Q	( Leen *et al.*, 2010) dbSNP rs779073410 significance unknown	affect protein function	probably damaging
	R92W	( Schneider *et al.*, 2009) GLUT1DS2 omim:612126	affect protein function	probably damaging
	R93Q	dbSNP rs80359815 significance unknown	tolerated	probably damaging
	R93W	( Joshi *et al.*, 2008) GLUT1DS2 omim:612126	affect protein function	probably damaging
	R333W	( Klepper & Voit, 2002; Klepper *et al.*, 2001) EIG12 omim:614847	affect protein function	probably damaging
	R334Q	ClinVar rs892715050 significance unknown	affect protein function	probably damaging
GLUT3	G89V	dbSNP rs758117298 significance unknown	affect protein function	probably damaging
	R90W	not reported in dbSNP ( rs1270428275 R90T )	affect protein function	probably damaging
	R91C	dbSNP rs756172777 significance unknown	affect protein function	benign
	R91H	dbSNP rs145936296 significance unknown	tolerated	benign
	R331K	dbSNP rs770855736 significance unknown	affect protein function	probably damaging
	R331S	dbSNP rs749200071 significance unknown	affect protein function	probably damaging
	R331W	not in dbSNP significance unknown	affect protein function	probably damaging

* References given to literature describing clinical appearance, OMIM entries, and dbSNP entries given, if available.

### Verification of constraining approach

**Figure 2.**
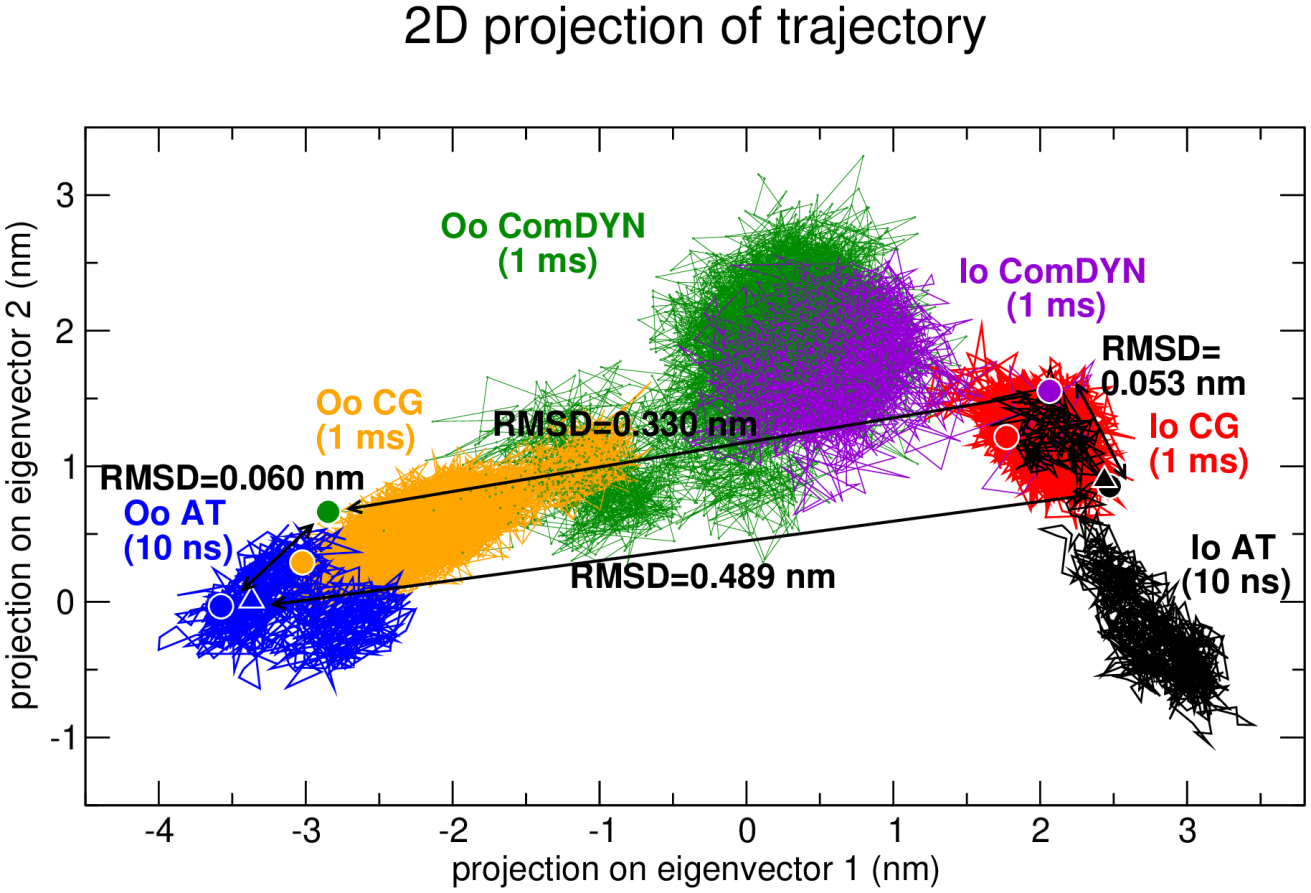
Two-dimensional essential dynamics plot of the simulations. In this projection, eigenvector 1 corresponds to changes from O _O_ (left) to I _O_ (right), showing also the overlap and differences between the AT (full atomistic), the CG (coarse-grained), and the ComDYN (common constraints CG) simulations. Note that there is considerable overlap in the sampling, but that the time-scale of the AT simulations only samples conformations around the I _O_ and O _O_ states and the elastic network in the CG simulations also limits the visited conformations, while the ComDYN samples a large number of conformations between both states.; I _O_, inward open state (PDB-ID: 5EQI); O _O_, outward occluded state (PDB-ID: 4ZW9). Spheres indicate the respective starting conformations for each method, triangles to show the corresponding crystal structures; black arrows and labels indicate the RMSD between the starting conformations of AT and ComDYN simulations.

Firstly, we want to verify if our common constraints-based approach for coarse-grained MD simulations (ComDYN) is able to sample the intermediate states between the I _O_ and O _O_ states. Including only elastic network constraints that differed less than 1Å between the I _O_ and O _O_ states in ComDyn, resulted in 1025 constraints (39.9% of the 2568 in the Martini elastic network) for the O _O_ state and 978 (42.2% of 2315) for the I _O_ state. We performed essential dynamics (ED) analysis ( Amadei *et al*., 1993; Van Aalten *et al*., 1997) to compare AT, traditional CG MARTINI, and ComDYN simulations, as described in the Methods. Figure 2 shows 2D plots of the first two (largest) ED eigenvectors, representing the extracted correlated motions over the ensemble of our simulations. The first eigenvector (EV1) represents the major conformational changes between the O _O_ and I _O_ states, as also indicated by the RMSD values between starting states. The changes observed along EV2 may also be genuinely part of the state transition, however we cannot exclude the possibility that some of these conformational changes may relate to differences between the forcefields used. The sampling of the different states, inward-open and outward-occluded, in AT simulation hardly converges due to their limited time scales. The regular CG simulations reach much longer timescales, and already sample more intermediate conformations, but there is no overlap. The ComDYN simulations, on the other hand, also sample many states intermediate to the inside-open and outside-open starting states, compared to the other simulations. This shows that improved sampling of large conformational transitions may be attained using this approach of CG and ComDyn simulations.

### Probing conformational changes

To probe the degree of the conformational changes during ComDyn simulations of the wild types and the mutants in more detail than done with the ED analysis, two distances were used to describe the opening and closing of the periplasmic and cytoplasmic sides of the transporter. Nagarathinam *et al*. (2018) studied a bacterial homolog of GLUT1 and GLUT3, and analysed the movement between TM5 and TM8. In the extended data, Figure S4 ( Feenstra, 2019a), we can see that the distributions obtained from our ComDYN simulations, resemble those reported by Nagarathinam *et al*. (2018), providing an independent validation that our ComDYN approach is able to sample biologically relevant conformational states for large scale motions, such as those involved in the glucose transporter mechanism. Nevertheless, there are differences between the distance distributions in our work and that of Nagarathinam *et al*. (2018), which could, apart from obvious differences comparing human glucose transporters with a bacterial multidrug transporter (see also extended data, Figure S4 and Table S3 ( Feenstra, 2019a)), also arise from differences in the sampling protocols applied in the two studies.

**Figure 3.**
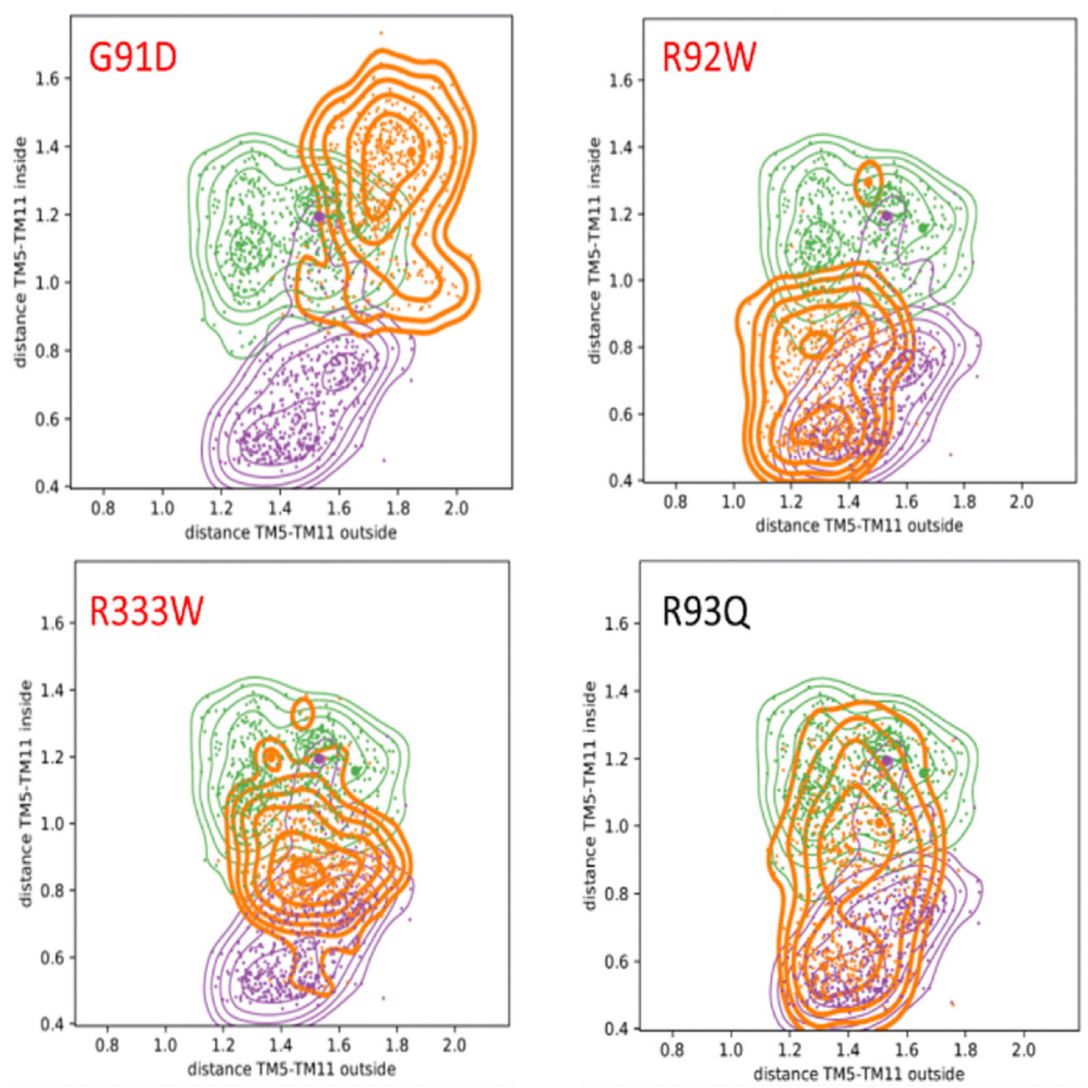
Distance plots of the inner and outer distances along the order parameter TM5-TM11 in nm over the complete time span of the CG ComDYN simulations (see Figure 1D). Colour code: wild types I _O_ in purple (PDB-ID: 5EQI), O _O _in green (PDB-ID: 4ZW9), mutants in orange. Pathogenic mutants are highlighted in red. It should be noted that in contrast to the benign R93Q mutant, the pathogenic mutants do not sample the I _O_ and O _O_ states during the simulation, which strongly indicates that the mutation blocks the proper opening and closing mechanism. Corresponding plots for all mutations are in the extended data, Figure S4. The corresponding quantification of these plots provided as shift and overlap are given in Table 2.

Therefore, in addition to the TM5-TM8 distances, we extended the analysis to other helices along and across the rim that make up for the entire SLC transporter architecture, allowing us to monitor changes in their position (see Figure 1C, D). For each of these order parameters, we calculated the distance at the inside and outside of the protein with respect to the membrane. Using this analysis, we can immediately observe the changes occurring between the inward-open and outward-occluded states. We see several distances changing significantly during this process: TM1/TM2(in), TM1/TM5(out), TM1/TM7(out), TM1/TM8(in), and TM5/TM11(in) are all closing, while TM1/TM2(out), TM1/TM5(in), TM1/TM7(in), TM2/TM11(in), TM2/TM8(out), and TM5/TM11(out) are opening (see extended data, Table S3 and Figure S4 for more details ( Feenstra, 2019a)); these motions are also schematically summarised in extended data, Figure S3 ( Feenstra, 2019a). Table 2 summarises the overlap and shift between the sampled distributions of distances between the wild type and each of the mutants for TM5 and TM11 that exhibited the strongest effects and conformational changes during the simulations (the complete table of the distributions for all order parameters are available in the extended data, Table S3 ( Feenstra, 2019a)). The corresponding conformational distributions from the ComDYN simulations, calculated as a function of the inner and outer distances between TM5 and TM11 are given in Figure 3.

**Table 2.** Overview of the changes in inside and outside distances in the CG ComDYN simulations, between transmembrane helix 5 (TM5) and TM11. Figure 3 shows the corresponding distance plots for some of the mutants. These are quantified using the fraction of overlap between both distributions in sampling distributions of sampled conformations, calculated as the integral of the minimum of both functions, i.e., the volume (normalized to a maximum of one), given in %, of the wild type and mutant (small value is large change), and the shift of the peak location (in nm including the direction; positive is to larger distances). The pathogenic mutations are underlined. All the large shifts (absolute above 0.15 nm) and small overlaps (below 0.6 nm) are set to bold. For a visual aid, the shifts are set in italic.

GLUT1		G91D	R333W	R334Q	R92Q	R92W	R93Q	R93W
Inside	Overlap	**4%**	**24%**	**47%**	60%	64%	**46%**	62%
	*Shift*	***0.84***	***0.34***	*-0.05*	*-0.02*	*-0.02*	***0.28***	*-0.03*
Outside	Overlap	**20%**	69%	70%	70%	**48%**	70%	61%
	*Shift*	***0.25*>**	*-0.13*	*-0.13*	*0.02*	***-0.23***	*-0.12*	***-0.18***
**GLUT3**		**G89V**	**R331K**	**R331S**	**R331W**	**R90W**	**R91C**	**R91H**
Inside	Overlap	**25%**	67%	**9%**	**7%**	**6%**	56%	**12%**
	*Shift*	***0.27***	*-0.06*	***-0.28***	***-0.35***	***-0.32***	*-0.12*	***0.30***
Outside	Overlap	**28%**	57%	50%	53%	50%	62%	**15%**
	*Shift*	***0.37***	*0.11*	*0.14*	***0.21***	*0.12*	***0.33***	***0.58***

### Impact of mutations on dynamics

Not all mutations have a high impact on the overall dynamics (extended data, Table S3 and Figure S4 ( Feenstra, 2019a)). However, in GLUT1, the reported pathogenic mutation G91D has a profound effect on the dynamics of the protein (i.e., a low overlap and large shift, see Table 2). Also when we consider the distance of TM5-TM11, as shown in Figure 3, the strongest effect is observed for the pathogenic G91D mutant: its distribution varies strongest from the wild types, and hardly visits the inward-open and outward-occluded states. Furthermore, Figure 3 shows that for the pathogenic R92W and R333W mutations, only one state or small parts from both can be accessed. For the benign mutant R93Q, in contrast, it can be seen that both states, inward-open and outward-occluded, are sampled thoroughly during the simulations. Assuming that the relative distance between the two helices is crucial for the correct functioning, this strongly suggests that the pathogenic mutations directly affect the opening and closing mechanism of the GLUT1 transporter.

Mutations in GLUT3 show similar behaviour in TM dynamics compared to those in GLUT1. Here, two mutants with strong abnormal behaviour can be identified: G89V and R91H ( Table 2; extended data Figure S4 ( Feenstra, 2019a) shows the corresponding distance distribution plots). Additionally, similar to the observations for pathogenic mutations on GLUT1, these mutations no longer sample intermediate states associated with the transport function, unlike the wild type and many of the other mutations. This strongly suggests that the corresponding mutations between GLUT1 and GLUT3 also have the same direct blocking effects on the opening and closing mechanism of the GLUT3 transporter. However, it should be noted that we cannot make any conclusions about the clinical significance of these GLUT3 mutants, as none have been reported to be pathogenic. A next step to elucidate the complete pathway would be to extend the analysis to the outward-open conformation, and include ligand-bound states ( Delemotte, 2019), which may shed further light on some of the currently still unexplained pathogenic mutations.

## Conclusion

Using extensive ComDYN simulations of GLUT1 and GLUT3 wild type and several clinically relevant mutations, we provide an effective way to study dynamic effects of mutations on the molecular mechanism of human glutamate transporter proteins. Without using full-atomistic details, we were able to get insight into the opening and closing mechanisms, which may account for the (dys)function of the SLC family caused by pathogenic mutations around the conserved RXGRR-motif. Through these mutations (especially G91D, R92W and R333W in GLUT1), we observe that the distances between TM5 and TM11 across the rim of the solute carrier structure are affected the strongest. For this reason, we chose them as our order parameter to explain the abnormal behaviour in the dynamics of the transporter opening and closing mechanism for some of the observed mutants. It should be mentioned that this does not provide an ultimate order parameter to explain all the effects of the pathogenic mutations, but allows us to better understand some of the effects caused by these pathogenic mutations. Comparing atomistic (AT), coarse-grained MARTINI (CG), and ComDYN simulations, our work shows that our CG ComDyn simulations are sufficiently accurate to sample the intermediate states between the conformational states and capture some of the effect of the mutations on the dynamic and function of these transporter proteins. This helps to elucidate the effects of pathogenic mutations on the structure and dynamics of the GLUT1 and GLUT3 transporters which were previously not understood.

## Data availability

### Underlying data

Crystal structures for GLUT1 I _O_ state ( Deng *et al*., 2015) and for GLUT3 O _O_ state ( Kapoor *et al*., 2016) were obtained from the Protein Data Bank, under accession numbers 5EQI and 4ZW9, respectively.

### Extended data

Open Science Framework: ComDYN. https://doi.org/10.17605/OSF.IO/F82H5 ( Feenstra, 2019a).

The following extended data are available:

- Data.tgz. data files accompanying analyses performed in this study.
- Table S1. Summary of the molecular composition of simulated systems.
- Table S2. Structurally conserved helical segments between 4ZW9 and 5EQI.
- Table S3. Wild-type and Mutant simulations compared by Overlap and shift between TM helix distance distributions.
- Figure S1. Sequence alignment between *E. coli* multi-drug transporter MDFA, and human glucose transporters GLUT1 and GLUT3.
- Figure S2. Pipe representation of the inward-open conformation of the transporter.
- Figure S3. Schematic view of the observed pore mechanism going from the inward-open state to the outward-occluded state.
- Figure S4. Distribution of inside and outside helix distances for all examined mutants in GLUT1 and GLUT3.

Extended data are available under the terms of the Creative Commons Zero "No rights reserved" data waiver (CC0 1.0 Public domain dedication).

## Software availability

**Scripts used to setup and analyze the ComDYN simulations available from:**

https://github.com/ibivu/ComDYN.

**Archived source code at time of publication:** https://doi.org/10.5281/zenodo.2591477 ( Feenstra, 2019b).

**License:** GNU General Public License 3.0.
