# Supplementary material for: Impact of pathogenic mutations of the GLUT1 glucose transporter on solute carrier dynamics using ComDYN enhanced sampling
Source: F1000Res. 2022 Jun 13;8:322. Originally published 2019 Mar 22. [Version 2] doi: 10.12688/f1000research.18553.2 (PMC11780263; doi:10.12688/f1000research.18553.2)
Supplement: Peer review history

# Reviewer report: Reviewer response for version 2

**DOI:** 10.5256/f1000research.133928.r358305

**Published:** 2025-01-29

**Authors:** Lívia Maria Ferreira Sobrinho

**Affiliations:** 1 Federal University of Minas Gerais, Minas Gerais, Brazil

General Summary of the Article

 This study presents an innovative exploration of conformational dynamics in the GLUT1 glucose transporter, utilizing coarse-grained simulations and the ComDYN technique. The research highlights the impact of specific mutations on the structure and dynamics of the transporter, with implications for clinical conditions such as GLUT1 deficiency syndrome. The study significantly contributes to understanding the dynamic behavior of transporters and proposes a promising methodology for future investigations.

 General Assessment

- Strengths:

1. Scientific Relevance: The topic is highly pertinent, considering the importance of GLUT1 in physiological processes and its association with genetic disorders.
2. Innovative Methodology: The introduction of the ComDYN technique demonstrates an efficient approach to exploring conformational transitions in transport proteins, providing a robust model for dynamic analysis.
3. Clarity and Organization: The article is well-structured, with figures and tables that enhance the understanding of the results, enabling readers to follow the scientific rationale effortlessly.
4. Methodological Contribution: The combination of coarse-grained simulations and dynamic analyses provides detailed insights into conformational transitions.
5. Significant Results: The analyses highlight the impact of specific mutations, such as G91D, providing clear evidence of dynamic alterations associated with pathogenicity.
6. Constructive Discussion: The discussion acknowledges methodological limitations and proposes clear directions for future studies, such as the inclusion of ligand-bound states and atomistic simulations.

- Minor Limitations:

1. Restricted Scope of Conformational States: While the study focuses on the "outward occluded" and "inward open" states, including additional states in future research could broaden insights into the conformational cycle.
2. Absence of Ligands: The choice to work with ligand-free states is valid for exploring intrinsic dynamics but integrating ligands could provide a more comprehensive perspective.

Detailed Comments

 1. Introduction

- Strengths:

The introduction offers a comprehensive overview of the importance of GLUT1 and the impact of pathogenic mutations. The explanation of the transporter's role in the conformational cycle is clear and well-founded.

- Suggestion: Briefly expand the introduction to discuss how the methodology could be applied to other SLC-type transporters.
- 2.  Methodology
- Strengths:

1. The use of the MARTINI forcefield is appropriate and enables efficient exploration of large-scale dynamics.
2. The justification for the 1 Å contact cutoff is consistent with the limitations of the coarse-grained model.
3. The choice of ligand-free simulations allows detailed analysis of the intrinsic dynamics of the transporter.

- Minor Limitation:
  Briefly discussing how alternative contact cutoffs could complement the results would be a valuable addition, though not essential.

3. Results

- Strengths:

1. The graphs and tables are clear and well-presented, providing visual support that facilitates data interpretation.
2. The analysis of TM5-TM11 distances is robust and demonstrates how mutations like G91D impact conformational dynamics.
3. The figures effectively describe transitions, and the captions link the results to the initial hypotheses.

4. Discussion and Conclusion

- Strengths:

1. The discussion effectively connects the results to the overall study objective, highlighting the impact of the analyzed mutations.
2. The conclusion is clear and acknowledges methodological limitations without undermining the scientific contribution of the work.
3. The proposal to expand the study to include ligand-bound states demonstrates a forward-looking approach to continuing the research.

Suggestions for Improvement

1. Future Additions: Explore additional conformational states and include ligands in future studies to complement the presented analyses.
2. Methodological Details: Provide a brief justification regarding the limitations of the contact cutoff to reinforce the methodological choices.

Is the work clearly and accurately presented and does it cite the current literature?

Yes

If applicable, is the statistical analysis and its interpretation appropriate?

Not applicable

Are all the source data underlying the results available to ensure full reproducibility?

Yes

Is the study design appropriate and is the work technically sound?

Yes

Are the conclusions drawn adequately supported by the results?

Yes

Are sufficient details of methods and analysis provided to allow replication by others?

Yes

Reviewer Expertise:

Clinical genetics; Inborn errors of metabolism; GLUT1 deficiency syndrome

I confirm that I have read this submission and believe that I have an appropriate level of expertise to confirm that it is of an acceptable scientific standard.

# Reviewer report: Reviewer response for version 2

**DOI:** 10.5256/f1000research.133928.r140632

**Published:** 2022-07-22

**Authors:** Lucie Delemotte

**Affiliations:** 1 Department of Applied Physics, Science for Life Laboratory (SciLifeLab), KTH Royal Institute of Technology, Stockholm, Sweden

The manuscript is improved and describes accurately the insights made possible by the newly developed ComDYN method.

 I noticed two small issues that should be resoved:

1. In the abstract, a reference is made to "both states of the protein". However, it should be clarified which two states given that this transporter cycles through more than 2 states during its conformational cycle.
2. In the conclusion, "human glutamate transporter proteins" should be replaced by "human glucose transporter proteins".

Is the work clearly and accurately presented and does it cite the current literature?

Yes

If applicable, is the statistical analysis and its interpretation appropriate?

Yes

Are all the source data underlying the results available to ensure full reproducibility?

Yes

Is the study design appropriate and is the work technically sound?

Partly

Are the conclusions drawn adequately supported by the results?

No

Are sufficient details of methods and analysis provided to allow replication by others?

Yes

Reviewer Expertise:

Membrane proteins, ion channels, molecular dynamics simulations, enhanced sampling

I confirm that I have read this submission and believe that I have an appropriate level of expertise to confirm that it is of an acceptable scientific standard.

# Reviewer report: Reviewer response for version 1

**DOI:** 10.5256/f1000research.20307.r47253

**Published:** 2019-05-08

**Authors:** Jocelyne Vreede

**Affiliations:** 1 Van 't Hoff Institute for Molecular Sciences, University of Amsterdam, Amsterdam, The Netherlands

The paper reports a molecular dynamics simulation study of the glucose transporter GLUT1 using all-atom and coarse grained force fields, in combination with a conserved elastic network. Using essential dynamics analysis and comparison of various distances the authors compared the dynamics of the wild type protein to pathogenic variants in the outward occluded and inward open states. This procedure enabled the prediction of the effect of mutations on the dynamics of GLUT1 and GLUT3. 

 The manuscript requires a few clarifications to improve my understanding:

- As I am not very familiar with the mechanism of glucose transporters, I would like a few sentences describing how these proteins work, thus giving more context to the different conformational states I _o_ and O _o_. Also in the conclusion, a bit more context as to how the states interconvert and the impact of the mutations on these transitions would aid my understanding tremendously. 
- The manuscript reports results on mutations at positions 92, 93, 333 and 334, which are highlighted in Fig. 1. Only mutations 92 and 93 are discussed in the introduction. For more context, the mutations at 333 and 334 should be discussed in the introduction as well. 
- What motivates the cut-off of 1 angstrom for including constraints in the elastic network? Would 1.5 angstrom or 2 angstrom work as well? 
- As essential dynamics analysis is performed on both all-atom and coarse grained simulations, I assume only C-alpha positions are included. Is this assumption correct? 
- What do the two eigenvectors shown in Fig. 2 mean? My interpretation is that EV1 is the transition from the O _o_ to the I _o_ state, and that EV2 is the transition from the all-atom to coarse grained-constrained description. If this interpretation is correct, would the conclusion be correct that the dynamics sampled in the different force fields overlap? 
- The O _o_ and I _o_ states as sampled with the consdyn and the AT approach seem quite different in Fig. 2. What could be the explanation for this difference? 
- Snapshots of the conformations at the maxima of the probability histograms would help my understanding of the differences as introduced by the mutations. 
- I do not understand how the overlap in the distributions in Table 2 is computed. 
- What is the unit of the shift in Table 2 and of the distances in Fig. 3?

Is the work clearly and accurately presented and does it cite the current literature?

Yes

If applicable, is the statistical analysis and its interpretation appropriate?

Partly

Are all the source data underlying the results available to ensure full reproducibility?

Yes

Is the study design appropriate and is the work technically sound?

Yes

Are the conclusions drawn adequately supported by the results?

Partly

Are sufficient details of methods and analysis provided to allow replication by others?

Yes

Reviewer Expertise:

Molecular simulation of proteins and DNA

I confirm that I have read this submission and believe that I have an appropriate level of expertise to confirm that it is of an acceptable scientific standard, however I have significant reservations, as outlined above.

# Response

**Published:** 2022-05-30

**Authors:** K. Anton Feenstra

**Affiliations:** VU University Amsterdam, The Netherlands

We thank the reviewer for the attention and time spent on our work, and respond to each of their comments in detail below:

1. Reply: We have added a short explanation of the basics of the SLC glucose transport and how the process cycles through the various states in the Introduction, and now also emphasize the main changes arising from the pathogenic mutations in the Conclusion.
2. Reply: We have added a discussion on the mutations at the R333 and R334 positions in the Introduction.
3. Reply: The 1Å cutoff retains 40% of the constraints, which is already relatively little. Shorter cut-offs would yield more restricted sampling. A wider cut-off would not necessarily provide better sampling, but would very likely lead to too large conformational changes or even unfolding of the transporter protein. This is now explicitly mentioned in the methods section under *Molecular Dynamics*.
4. Reply: Indeed, the ED analysis uses C-alpha for AT and backbone particles at C-alpha position for CG. We have now clarified this in the *Analysis of transporter dynamics* section in the Methods.
5. Reply: Indeed, transitions between Oo and Io are visible on EV1 in Figure 2; we have now added this explicitly in the figure caption. The sampled states do overlap between the different forcefield approaches. Moreover, as we also discuss in our reply to question 3 of Reviewer 2, the CG sampling partly overlaps the AT sampling on both sides of the Oo to Io transition, and partly with the ComDYN sampling more to the middle of the transition. Furthermore, the Oo ComDYN sampling overlaps considerably with the Io ComDYN sampling. We have also added the starting conformations in Fig 2 as an aid to the reader to better navigate this projected sampling space. We cannot exclude that EV2 may represent conformational changes due to differences between the forcefields, however as we are also sampling far longer timescales in the CG and ComDYN simulations, these differences may also genuinely be part of the transition between the Oo and Io states.
6. Reply: Both ComDYN simulations start at or near the conformations from the AT simulations, but this is not quite visible in the plot in Fig 2. We have now added spheres to indicate the respective starting conformations, as well as triangles to show the corresponding crystal structures. The CG simulations sample much longer timescales, so allow conformational transition that cannot be reached during the sampling time of the AT simulations. We have now clarified this in results, Section “Verification of constraining approach”.
7. Reply: The outcome of such a visualization would not be informative for a protein of this size. The differences in conformation (namely the distances between the transmembrane helices) are small and therefore difficult to visualize. Superpositioning snapshot does therefore not help to understand the differences occurring through the mutations. To capture molecular motions during our simulations, we use the defined inter-helix distances (cf. Figure 1D.) as order parameters, which are directly interpretable. 
8. Reply: ‘Overlap’ is the fraction of overlap between both distributions of sampled conformations, calculated as the integral of the minimum of both functions, i.e., the volume (normalized to a maximum of one) that represents the amount of sampling between two distributions. We have now clarified this in the section *Analysis of transporter dynamics* in Methods, with a reference to the relevant python script and now provide the ‘overlap’ in percentage to make this explicit.
9. Reply: They are all in nanometers (nm), we have added this now explicitly to the table and figure captions.

# Reviewer report: Reviewer response for version 1

**DOI:** 10.5256/f1000research.20307.r46135

**Published:** 2019-04-15

**Authors:** Lucie Delemotte

**Affiliations:** 1 Department of Applied Physics, Science for Life Laboratory (SciLifeLab), KTH Royal Institute of Technology, Stockholm, Sweden

This paper describes a computational study of the effect of mutations on GLUT transporter dynamics. The work consists of atomistic and coarse grained simulations, including some using a protocol called ConsDYN which imposes constraints on distances conserved across conformational transitions. The study identifies that several pathogenic and other mutations modify the structural ensemble visited by the protein.

 While the topic is important and computational methods are well-suited to answer the question, I have reservations about the study design and the conclusions reached:

  

1. A pathogenic mutation modifies the function of the protein such that cellular and organism function are altered. Glucose transporters carry out their function, i.e. importing sugars, via an alternating access cycle in which the transporter transits between outward-open and inward-open states via occluded states; whereas sugar binding from the extracellular medium promotes a transition to the inward facing state, sugar release to the inside intracellular medium promotes a return to the outward facing state. Other than the intrinsic dynamics of interconversion between states, the fact that the sugar modifies the stability of states and the kinetics of interconversion is key for function. Thus pathogenicity of a mutation could be due to many factors: among others, sugar binding, unbinding, (de)stabilization of one or more states on the functional cycle, modification of the rate of conversion between states, in the presence and/or absence of sugar. In this paper, the authors investigate the effect of mutations on the dynamics of interconversion between states. I believe the assumption should be spelled out more clearly, and the omission of all other possible effects on the sugar transport cycle should be explained.
  Relatedly, were the simulations performed in the presence or absence of sugar? Comparing both cases could lead to increased insights.
2. Why was the analysis limited to outward occluded and inward open states, when high-resolution structures of other states are available? If only two states should be considered, why not consider the inward open and outward open since they are the two end-points of the transport cycle?
3. The ConsDYN method can be an interesting way to promote conversion between states using coarse grained simulations. However, whereas the stated aim on p5 is “to check that ConsDYN is able to sample both I _o_ and O _o_ states”, Figure 2 reveals that ConsDYN simulations only sample intermediate states, instead of bridging between states. I would thus disagree with the conclusion according to which the method allows to capture the “conserved dynamics” and to “sample between the conformational states”. 
  Would lowering the force constant of the constraints imposed lead to further exploration of the landscape?
  The method also seems to have a serious conceptual drawback, in that it assumes that when switching from a state to another, common contacts are conserved, and additional ones are formed in either states. This does not seem to be a general feature of conformational changes in biological molecules and should be discussed.
4. Table 2 reports changes in distance between helices in the presence and absence of mutations, including pathogenic ones. Whereas the pathogenic G91D seems to cause major changes to the dynamics of the transporter, the other pathogenic mutations only alter some of the distance distributions. It does not appear that applying this methodology and measuring the difference in distance distributions as is done in Table 2 allows to predict pathogenicity. I thus disagree with the conclusions: “the distances between TM5 and TM11 can be used as order parameters to elucidate abnormal behavior in the dynamics of the transporter” and “ConsDYN simulations capture the effect of the mutations on the dynamic and function of the transporter proteins”.

Minor comments: 

1. The authors refer to a “channel” in the title and later in the manuscript. Do they mean the transporter lumen? In an alternating access mechanism, a channel is never observed.
2. p5: The difference between Nagarathinam *et al.* (2018) and this work is ascribed to differences in the transporter protein studied (bacterial vs human) but the authors cannot rule out that the differences can come from differences in the sampling protocol.
3. Were the CG or AA simulations of I _o_ and O _o_ analyzed in Figure 3, Table 2 and SI figures?
4. p6: “outer distances between TM5 and TM7 are given in Figure 3” should be replaced by “outer distances between TM5 and TM11 are given in Figure 3”.

Is the work clearly and accurately presented and does it cite the current literature?

Yes

If applicable, is the statistical analysis and its interpretation appropriate?

Yes

Are all the source data underlying the results available to ensure full reproducibility?

Yes

Is the study design appropriate and is the work technically sound?

Partly

Are the conclusions drawn adequately supported by the results?

No

Are sufficient details of methods and analysis provided to allow replication by others?

Yes

Reviewer Expertise:

Membrane proteins, ion channels, molecular dynamics simulations, enhanced sampling

I confirm that I have read this submission and believe that I have an appropriate level of expertise to state that I do not consider it to be of an acceptable scientific standard, for reasons outlined above.

# Response

**Published:** 2022-05-30

**Authors:** K. Anton Feenstra

**Affiliations:** VU University Amsterdam, The Netherlands

We thank the reviewer for the attention and time spent on our work, and respond to each of their comments in detail below:

1. Reply: All the simulations were carried out without glucose or any other ligand, we only consider the ligand-free states of the transporters. This implies that the pathogenicity of a mutant might already be stated through the intrinsic dynamics of the transporter without explicitly considering the effect of the mutation in the ligand-bound state. This does indeed not exclude that pathogenicity linked to a specific mutant might arise from a dysfunction of the binding mechanism. In this study we focus on the first part of the transport mechanism of SLC transporters, and introduce the ComDYN method, which is designed to capture conformational changes between different states. We have now added this point to the manuscript and explicitly mention that, to guarantee the study of all the relevant effects, simulations including the ligand-bound states might yield additional insight on the structure and dynamic of different mutants. However, this would only be feasible using full-atomistic simulations, since sugars are not well parametrized in the Martini force field. As this would pose strong limits on the time scales we could practically attain in our simulations, it is out of the scope of the current study.
2. Reply: We want to focus on the first part of the transport mechanism: moving from the inward open to the outward occluded state. The next step to elucidate the complete pathway will be to extend this to the outward-open conformation which is available in the PDB. For the present work, we focus on the impact of some mutations on the intrinsic dynamics of the protein (without considering the impact of any ligand) and show in this case study that ComDYN is able to sample these large conformational changes between states. We have now clarified this point in the manuscript.
3. Reply: We mostly agree, we have rephrased “sample both IO and OO states” to “sample the intermediate states between IO and OO states” throughout the manuscript.
4. Reply: A lower force constant would indeed lead to more sampling, just like a wider cut-off which we discuss in our response to question 3 of Reviewer 1, but it would not necessarily lead to a better sampling or better exploration of the landscape. At low force constants for the constraints, in the MARTINI forcefield it becomes increasingly likely that too large conformational changes become permissible, even up to the point of unfolding of the transporter proteins.
5. Reply: The elastic network used in the MARTINI forcefield captures all residue contacts in the tertiary structure. In the case of SLC about 40% of contacts are conserved between both states, in our definition of less than 1 Å difference in the elastic network distance. Except in the case of (complete) unfolding, the vast majority of these contacts will exist in multiple conformational states of a solute carrier protein. However, it should be noted that we used the word ‘conserved’ in two different meanings: the specific evolutionary conservation, and the more general meaning of preservation, in this case of the contacts between two conformational states. We now clarified this throughout the text by referring to ‘common constraints’, and also changed the name ConsDYN to ComDYN accordingly.
6. Reply: Indeed, from the dynamics observed we cannot predict pathogenicity. However, we can ‘elucidate’ the known pathogenicity, in the sense of clarifying where previously effects were not understood. We have made the limitations of the method more explicit in the manuscript. In particular, this sentence now reads “For this reason, we chose them as our order parameter to explain the abnormal behavior in the dynamics of the transporter opening and closing mechanism for some of the observed mutants.” In future approaches, sugar binding will certainly need to be considered in order to fully understand the altered dynamics and behavior of the pathogenic mutants.

Minor comments:

1. Reply: Indeed, we have now changed the title and appropriate sections in the text to refer to ‘transporter’ in stead of ‘channel’.
2. Reply: Indeed we cannot. We have clarified this in the text.
3. Reply: The CG ComDYN simulations are analyzed in Figure 3, Table 2 and SI figures. We now mention this explicitly in the captions of Fig 3 and Table 2 and Figure S3.
4. Reply: We thank the reviewer for pointing out this mistake, which we have now corrected.
